# Effects of a Multi-Component Nutraceutical (NUT2) on the Lipid Profile in Adults with Low-to-Moderate Cardiovascular Risk: A Randomized Placebo-Controlled Trial

**DOI:** 10.3390/nu18162632

**Published:** 2026-08-12

**Authors:** Vincenzo De Leo, Claudio Citterio, Adam B. Smith, Neil Fawkes

**Affiliations:** 1 Colledoro Medical Center, 53100 Siena, Italy; 2 Cooper Consumer Health, 75116 Paris, France; claudio.citterio@cooperconsumerhealth.com (C.C.); neil.fawkes@cooperconsumerhealth.com (N.F.); 3 Leeds Institute of Rheumatic & Musculoskeletal Medicine, University of Leeds, Leeds LS7 4SA, UK; a.b.smith@leeds.ac.uk

**Keywords:** nutraceuticals, LDL-C, berberine, bergamot, dyslipidaemia, metabolic syndrome, insulin resistance, homocysteine, cardiovascular risk, dietary supplementation

## Abstract

**Background:** Lifestyle intervention is recommended as first-line management for individuals with low-to-moderate cardiovascular (CV) risk and elevated low-density lipoprotein cholesterol (LDL-C), although dietary intervention alone often produces only modest lipid reductions. NUT2 is a proprietary multi-component nutraceutical formulated to target complementary pathways involved in lipid metabolism, oxidative stress and cardiometabolic risk. This study evaluated its efficacy and safety as an adjunct to dietary intervention. **Methods:** In this randomized, double-blind, placebo-controlled, parallel-group study, 90 adults with low-to-moderate CV risk and elevated LDL-C were randomized to receive NUT2 (*n* = 45) or placebo (*n* = 45) for 12 weeks, together with standardized dietary advice. The primary endpoint was a change in LDL-C at Week 12. Secondary outcomes included changes in total cholesterol, triglycerides, fasting glucose, fasting insulin, high-sensitivity C-reactive protein (hsCRP), homocysteine, anthropometric parameters, blood pressure and metabolic syndrome prevalence. **Results:** Baseline characteristics were generally comparable, although baseline lipid values differed between groups and were accounted for using baseline-corrected change scores. At Week 12, LDL-C decreased by 13.2% with NUT2 and increased by 1.6% with placebo; the baseline-corrected between-group difference was −23 mg/dL (95% CI −29 to −16; *p* < 0.001). Total cholesterol was also significantly reduced (corrected difference −26 mg/dL, 95% CI −36 to −17; *p* < 0.001). Significant reductions in homocysteine and lower fasting insulin concentrations were observed with NUT2. Metabolic syndrome prevalence was 13.3% with NUT2 versus 37.8% with placebo at Week 12 (*p* = 0.008), although this exploratory finding was based on small participant numbers. No significant between-group difference was observed for hsCRP. NUT2 was well tolerated, with no serious treatment-related adverse events. **Conclusions:** In adults with low-to-moderate cardiovascular risk whose LDL-C remained suboptimal despite dietary intervention, NUT2 produced significant and clinically relevant improvements in LDL-C and total cholesterol over 12 weeks. The significant reduction in homocysteine further supports the broader biological activity of the multi-component formulation. Favourable changes in insulin and metabolic syndrome status suggest potential additional metabolic effects, although these findings warrant further investigation in larger studies specifically designed for populations with metabolic dysfunction. NUT2 was safe and well tolerated. (Clinical trial registry: NCT07492264; trial registration number: NCT07492264, registration date: 23 March 2026.)

## 1. Introduction

Cardiovascular (CV) disease remains the leading cause of morbidity and mortality worldwide, driven largely by cumulative exposure to atherogenic lipoproteins across the life course. Contemporary ESC/EAS guidance [1], reflecting the evolution of European prevention strategies over the past decade, increasingly recognises cardiovascular risk as a continuum and places increasing emphasis on earlier intervention to reduce lifetime exposure to atherogenic lipoproteins.

In individuals with low-to-moderate estimated CV risk, current guidance continues to recommend lifestyle intervention as first-line management, including dietary modification, physical activity and weight optimisation, with pharmacological lipid-lowering reserved for those who fail to achieve guideline-defined targets or whose risk profile escalates. However, successive guideline updates have also acknowledged a growing clinical grey zone in patients with persistently elevated LDL-C who do not yet warrant statin therapy, but in whom inaction risks prolonged exposure to suboptimal lipid levels.

Despite being foundational, lifestyle interventions alone typically produce only modest reductions in LDL-C. Meta-analyses and systematic reviews consistently demonstrate mean LDL-C reductions of approximately 0.15–0.20 mmol/L with dietary advice alone [2], which is often insufficient for a substantial proportion of patients with dyslipidaemia to achieve recommended targets. This gap between what is advised and what is realistically achievable contributes to therapeutic inertia, poor adherence and clinician frustration, particularly in primary prevention settings.

Compounding this challenge is the heterogeneity of lipid phenotypes within the low–moderate cardiovascular risk population. Many individuals do not present with isolated hypercholesterolaemia but instead exhibit features of metabolic dysfunction, including insulin resistance, central adiposity, hypertriglyceridaemia and low-grade systemic inflammation, resulting in mixed dyslipidaemia rather than a single lipid abnormality [1,3]. In such patients, LDL-cholesterol concentrations may be only mildly elevated or “borderline” and may therefore underestimate overall cardiometabolic risk, which is amplified by the clustering of metabolic and inflammatory abnormalities [4,5]. Importantly, lifestyle modification alone in this group is frequently difficult to sustain and is associated with variable and often modest effects on lipid parameters, even under structured intervention conditions [6,7].

Despite their inclusion in European prevention guidelines, the role of nutraceuticals in lipid management remains poorly defined in routine clinical practice. Although ESC/EAS guidance acknowledges their optional use in selected low–risk individuals, healthcare professionals frequently lack clarity regarding when, in whom and for what objective such interventions should be applied. This uncertainty reflects a fragmented evidence base, variable product quality and the extrapolation of weak or non-clinical data, resulting in nutraceuticals being either dismissed outright or used indiscriminately rather than positioned as targeted, evidence-based adjuncts to lifestyle intervention.

NUT2 was developed and evaluated as a fixed-dose, multi-component formulation. Accordingly, the present study was designed to assess the efficacy and safety of the complete formulation as intended for use, rather than to quantify the independent contribution of each component. Although this design does not permit component-specific effects or synergistic interactions to be established, the known biological activities of the individual components provide the scientific rationale for their combined use and support the biological plausibility of the observed findings.

At the same time, growing evidence highlights that cardiometabolic risk in many low–moderate risk individuals is not driven by LDL-cholesterol alone but by the convergence of metabolic dysfunction, oxidative stress and low-grade systemic inflammation. Biomarkers such as high-sensitivity C-reactive protein (hsCRP) predict cardiovascular risk independently of LDL-C, underscoring the limitations of single-pathway or purely lipid-centric approaches in this population.

These considerations support a multi-target strategy, in which combinations of bioactive compounds address complementary and interrelated mechanisms underlying dyslipidaemia and metabolic risk. In contrast to single-ingredient interventions, rationally designed combinations may offer greater potential to optimise lipid profiles while simultaneously modulating associated metabolic and inflammatory pathways.

Against this background, Meda Pharma S.p.A. developed a patented, proprietary nutraceutical combination (NUT2) containing artichoke extract, bergamot polyphenols, berberine, folic acid, astaxanthin and chromium picolinate. Each component was selected to target distinct but interconnected pathways involved in lipid metabolism, glucose regulation, oxidative stress and homocysteine homeostasis, reflecting the multifactorial nature of cardiometabolic risk in contemporary primary prevention.

Unlike many previously studied lipid-lowering nutraceutical combinations whose principal activity is derived from red yeast rice and monacolin K, NUT2 is a monacolin-free, fixed-dose formulation combining artichoke extract, bergamot polyphenols, berberine, folic acid, astaxanthin and chromium picolinate. These components were selected on the basis of established or potential biological activities relevant to lipid metabolism, homocysteine regulation, oxidative stress and glucose homeostasis. The present study evaluated the efficacy and safety of the complete NUT2 formulation as intended for use and was not designed to determine the independent contribution of individual components or demonstrate synergistic interactions.

Although LDL-C reduction was the primary objective, secondary outcomes were prospectively assessed to characterise the broader biological and clinical effects of the fixed-dose formulation. These included measures of lipid metabolism, homocysteine regulation, glucose homeostasis and systemic inflammation, reflecting the established or potential biological activities of its components. Interpretation of individual secondary outcomes takes account of their prespecified status, statistical robustness and the limitations of the corresponding analyses.

The current study evaluated NUT2 as an adjunct to dietary intervention in adults with LDL-C concentrations between 115 and 190 mg/dL and an estimated 10-year risk of fatal cardiovascular disease of 1% to 5%, calculated using the contemporaneous European SCORE system. Participants did not have an indication for pharmacological lipid-lowering therapy under the 2012 ESC/EAS recommendations applied when the study was designed.

The present study therefore evaluated whether NUT2, used as an adjunct to dietary intervention, could improve plasma lipid parameters in adults with low-to-moderate cardiovascular risk, defined according to the European SCORE algorithm (estimated 10-year cardiovascular risk 1–5%) and contemporaneous ESC/EAS recommendations, who had LDL-cholesterol concentrations between 115 and 190 mg/dL and did not yet meet criteria for pharmacological lipid-lowering therapy.

## 2. Materials and Methods

### 2.1. Study Design

A randomized, parallel-group, longitudinal, placebo-controlled study was conducted to evaluate the efficacy of the NUT2 nutraceutical formulation (Meda Pharma S.p.A., Milan, Italy) added to the diet versus placebo (P) added to the diet. Participants were allocated in a 1:1 ratio to receive either NUT2 or the placebo for 12 weeks, in addition to standardized dietary recommendations. The trial adhered to the ethical principles of the Declaration of Helsinki, and all participants provided written informed consent prior to enrolment. Subjects retained the right to withdraw at any time without justification. There was no patient or public involvement in the design, conduct or reporting of the trial. This study was retrospectively registered with Clinicaltrials.gov prior to publication (trial registration number: NCT07492264, registration date: 23 March 2026). Ethics approval was granted by Comitato Etico Indipendente (CEI), Istituto di Ricerche Cliniche e Bioingegneria, Milan, Italy (Reference: E1419; date of approval 13 January 2020).

### 2.2. Trial Setting

The trial was conducted at two tertiary care medical centres in Siena, Italy, between 21 February 2020 and 21 December 2020.

### 2.3. Participants

#### 2.3.1. Inclusion Criteria

Eligible participants met all of the following criteria: written informed consent and willingness to comply with study procedures; ability to communicate effectively and follow study requirements; male or female, aged 18–70 years; LDL-C plasma concentrations > 115 mg/dL and <190 mg/dL; triglycerides < 400 mg/dL; estimated 10-year cardiovascular risk between 1% and 5% based on SCORE charts; not requiring pharmacological lipid-lowering therapy according to the 2012 ESC/EAS guidelines; coverage under the national Social Security health system; negative pregnancy test where applicable.

#### 2.3.2. Exclusion Criteria

Participants were excluded if they met any of the following conditions: presence of cardiovascular disease or estimated 10-year CV risk > 5%; obesity (BMI > 30 kg/m^2^) or diabetes mellitus; current use of lipid-lowering medications, lipid-modulating supplements or drugs affecting lipid metabolism; antihypertensive treatment not stable for at least 3 months; anticoagulant therapy or uncontrolled hypertension (SBP > 190 mmHg or DBP > 100 mmHg); known thyroid, gastrointestinal or hepatobiliary disorders; any medical or surgical condition that could impair adherence to the study protocol; current or past alcohol or drug abuse; malignancy within the previous 5 years; history or clinical evidence of chronic inflammatory conditions (e.g., severe arthritis, systemic lupus erythematosus) or current use of immunosuppressive agents/long-term glucocorticoids; any significant concomitant disease compromising safety or study completion; known hypersensitivity to any component of the investigational product; women of childbearing potential not using reliable contraception, pregnancy or breastfeeding.

### 2.4. Sample Size Determination

Sample size was calculated based on prior literature. To detect a 15 mg/dL difference (SD 20 mg/dL) in LDL-C reduction between groups after 3 months, with 90% power and a two-tailed significance level of 5%, 39 subjects per group were required. Allowing for a 13% dropout rate, 45 participants were randomized to each study arm, for a total of 90 subjects.

### 2.5. Intervention

The investigational product (NUT2) was a patented proprietary combination of natural-origin nutraceuticals comprising: artichoke extract 100 mg, bergamot extract 300 mg (equivalent to flavonoids 120 mg), berberis aristata extract 588 mg, astaxanthin 0.50 mg, folic acid 0.20 mg and chromium 0.04 mg. Participants assigned to the intervention group consumed one tablet daily for 12 weeks.

### 2.6. Comparator

The comparator was a placebo (“P”) composed only of excipients and colorants, identical in appearance and packaging to NUT2 but without the natural active ingredients. All participants, irrespective of treatment allocation, received standardized dietary counselling recommending a low-glucose, low-calorie, low-fat diet tailored to individual clinical conditions.

### 2.7. Randomisation

After pre-screening, subjects meeting the inclusion and exclusion criteria were advised to follow a low-cholesterol (<200 mg/day) diet for a 28-day (±3 days) run-in period (“standard care”, SC). Following the run-in period, dietary adherence was assessed using participant-completed dietary questionnaires and self-reported compliance. Participants reporting ≥80% adherence to the prescribed diet and demonstrating a <10% reduction in LDL-cholesterol during the run-in period were considered eligible for randomisation. Eligible participants were randomised in a 1:1 ratio to receive either NUT2 plus standard care (NUT2+SC) or placebo plus standard care (P+SC). The allocation sequence was generated by a designated representative of the sponsor using an online block randomisation tool and implemented through a pre-prepared double-blind randomisation schedule provided to each study centre. Allocation concealment was ensured during product packaging by assigning a unique serial number to each study product corresponding to the treatment assignment specified in the randomisation schedule. The allocation code was retained by the sponsor within the Trial Master File and remained concealed until completion of data entry from the paper case report forms into the study database. Participants and investigators remained blinded to treatment allocation throughout the study. Emergency unblinding was permitted only when knowledge of the assigned treatment was considered essential for the clinical management of an individual participant.

### 2.8. Objectives

#### 2.8.1. Primary Objective

To compare the reduction in LDL-C levels after 12 weeks of treatment between the NUT2 and placebo groups.

#### 2.8.2. Secondary Objectives

Secondary endpoints included the effects of NUT2 versus placebo on: LDL-C after 6 weeks; homocysteine concentrations; fasting glucose and insulin; high-sensitivity C-reactive protein (hsCRP); anthropometric measures, including waist circumference and body weight; blood pressure; metabolic syndrome; short-term tolerability and acceptability.

At study completion, participants rated their willingness to continue treatment (Yes/No). A principal investigator evaluation of response to treatment was also provided on a 5-point scale: Null, Poor, Moderate, Good and Excellent.

### 2.9. Measurements and Procedures

Fasting plasma glucose and lipid profiles were analysed using standardized laboratory methods. Waist circumference was measured at each visit midway between the lowest rib and the iliac crest using a flexible anthropometric tape.

Blood pressure (BP) was measured according to European Society of Hypertension/European Society of Cardiology guidelines (ESH/ESC 2007) [8]. After 5 min of seated rest, three BP measurements were obtained at 2 min intervals using a standard sphygmomanometer. The average of the three readings was used for analysis.

Metabolic syndrome was defined as the presence of at least 3 out of the following 5 factors: increased arterial pressure (≥130/85 mmHg); serum triglycerides ≥ 150 mg/dL; serum HDL cholesterol, males ≤ 40 mg/dL, females ≤ 50 mg/dL; waist circumference, males ≥ 104 cm, females ≥ 88 cm; elevated fasting glycaemia ≥ 100 mg/dL. Metabolic syndrome was assessed at baseline and 12 weeks.

Adverse events, intercurrent illnesses and compliance were assessed every 6 weeks during active treatment.

### 2.10. Statistical Analysis

The study protocol prospectively defined both a Full Analysis Set (FAS) and a Per Protocol (PP) population. The FAS comprised all randomised participants who received at least one dose of study treatment and had at least one post-baseline efficacy assessment, while the PP population excluded participants with major protocol deviations. As all randomised participants completed the study without major protocol deviations, the FAS and PP populations were identical, and all analyses were therefore performed on the complete randomised cohort. No imputation of missing data was required because no efficacy outcome data were missing.

A full descriptive analysis was performed for all continuous parameters, including the number of observations (N), mean and standard deviation (SD). Categorical variables were summarized using absolute and relative frequency distributions.

Baseline homogeneity between treatment groups and the assessment of clinical efficacy were evaluated using the χ^2^ test or Fisher’s exact test for categorical variables. Differences between the arms were evaluated using the Wilcoxon rank sum test and mixed analysis of variance (MANOVA, between and repeated measures). All efficacy analyses were corrected for baseline values, consistent with the pre-specified statistical analysis plan, to account for any baseline differences between treatment groups. Specifically, change scores (follow-up minus baseline) were analysed using mixed repeated-measures analysis of variance (ANOVA), with treatment group (NUT2 vs. placebo) as the between-subject factor and study visit (Week 6 and Week 12) as the within-subject factor. The primary comparison was the treatment effect over time, including the treatment-by-visit interaction where appropriate.

All statistical tests were two-tailed with an α-level of 0.05. Statistical analyses were performed using R, version 4.5.1.

## 3. Results

### 3.1. Participants

**Figure 1:**
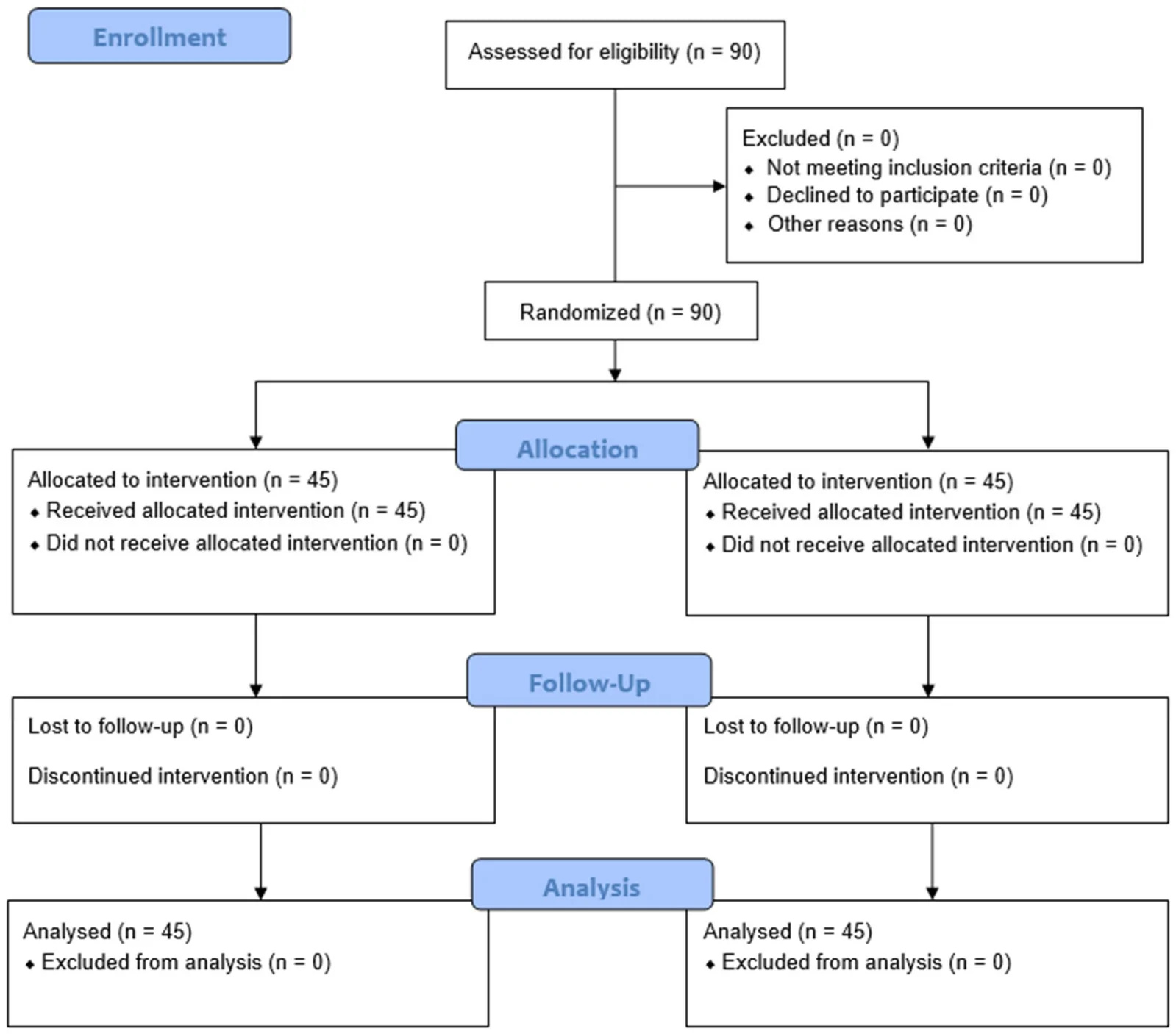
CONSORT participant flow diagram.

The CONSORT participant flow diagram is shown in Figure 1. A total of 90 dyslipidemic subjects were randomized to receive either NUT2 (*n* = 45) or placebo (*n* = 45). Baseline demographic and lifestyle characteristics—including age, sex distribution, BMI, smoking status, family history, physical activity and alcohol consumption—were comparable between groups. Waist circumference was slightly higher (approx. 4 cm difference) in the placebo group (*p* = 0.03) (Table 1).

### 3.2. Baseline Clinical Parameters

Baseline clinical variables, including systolic and diastolic blood pressure, heart rate, total cholesterol (Tot-Chol), LDL-C, HDL-C, triglycerides (TG), fasting plasma glucose, body weight and prevalence of metabolic syndrome, were similar between treatment arms, although there were some statistically significant differences (Table 2). Mean weight was slightly greater in the placebo arm (*p* = 0.3).

For instance, systolic blood pressure was marginally lower for the NUT2 arm (*p* = 0.042), whereas heart rate was higher (*p* = 0.005) at baseline. For the baseline lipid values (Table 2), Tot-Chol (*p* = 0.006) and LDL-C (*p* = 0.003) levels were higher at baseline for NUT2 relative to placebo. Conversely, baseline TGs were lower for the NUT2 arm (*p* = 0.042). No statistically significant differences were observed between the two arms for HDL-C, fasting plasma glucose (glycaemia) and homocysteine. Although a higher proportion of participants with metabolic syndrome were present in the placebo arm (29% versus 18% in the NUT2 arm), this was not statistically significant (*p* = 0.2).

The mean serum insulin level was slightly higher for the NUT2 arm (*p* = 0.3); mean hsCRP levels were higher for the placebo arm (*p* = 0.4).

### 3.3. Changes at 6- and 12-Week Follow-Up

#### 3.3.1. Lipid Parameters

There were statistically significant changes over time within each study arm for all lipid parameters with the exception of the triglyceride and glycaemia parameters (Table 3).

**Figure 2:**
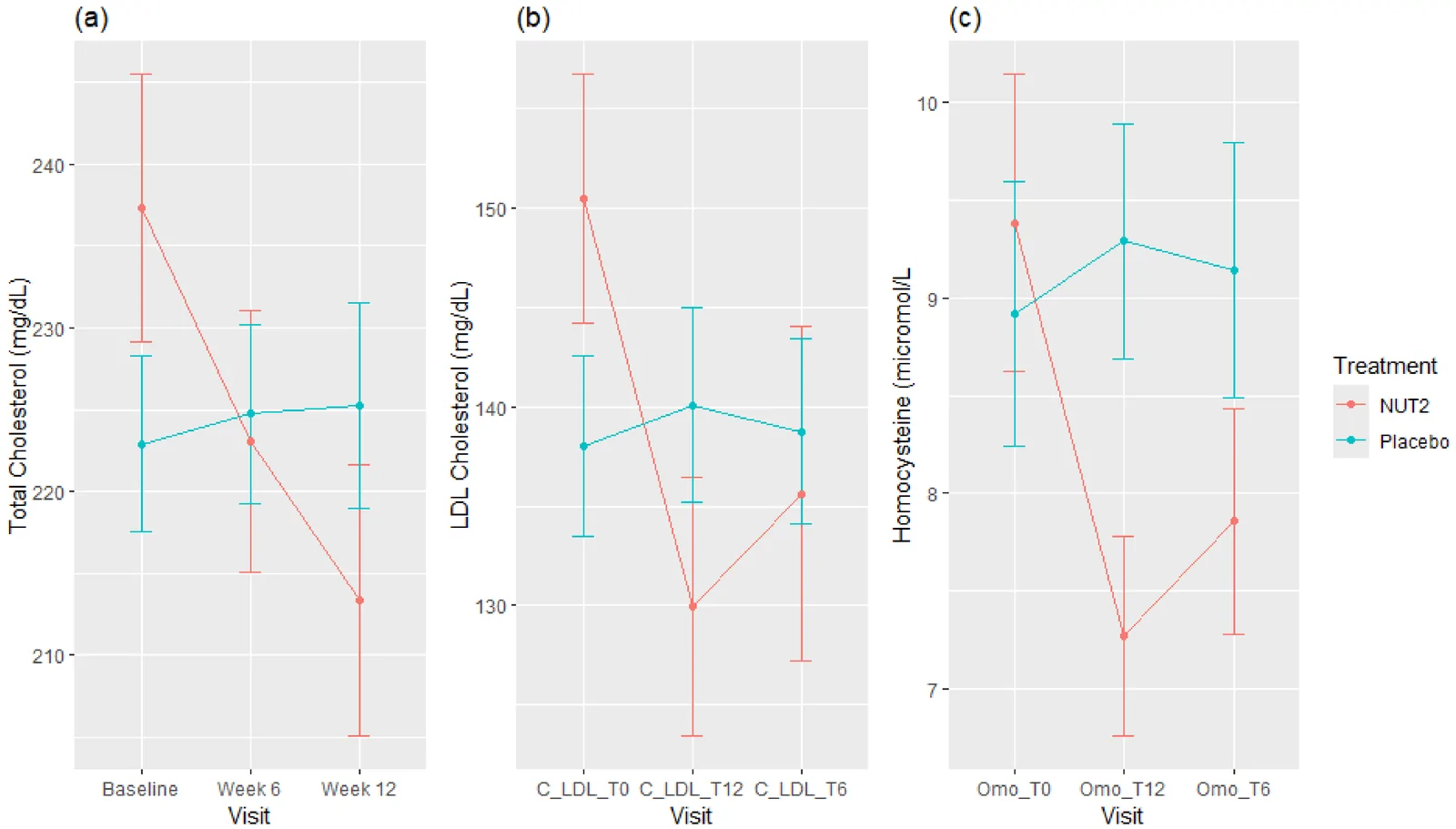
Lipid parameters by study visit. (**a**) Total cholesterol; (**b**) LDL cholesterol; (**c**) homocysteine.

In terms of the differences between the two arms at the follow-up visits, at 6 and 12 weeks, NUT2 supplementation produced significant reductions in Tot-Chol compared with baseline (−6.0% and −10.1%, respectively), whereas Tot-Chol slightly increased in the placebo group (0.8% and 1.1%). The between-group difference at 12 weeks was statistically significant (*p* = 0.002) (Figure 2a, Table 3).

LDL-C concentrations showed similar trends (Figure 2b). NUT2 resulted in reductions of −9.9% at week 6 and −13.2% at week 12, whereas LDL-C increased in the placebo group (+0.6% and +1.6%). This difference was statistically significant at week 12 (*p* = 0.005). Given the baseline imbalance in total cholesterol and LDL-C between groups, the primary lipid analysis was performed with adjustment for baseline values, consistent with standard RCT methodology for trials with pre-existing covariate imbalance. Adjusted analyses (i.e., baseline-corrected change scores) confirmed highly significant between-group differences for both total cholesterol (−26 mg/dL, 95% CI −36, −17, *p* < 0.001 at 12 weeks) and LDL-C (−23 mg/dL, 95% CI −29, −16, *p* < 0.001 at 12 weeks), as well as for triglycerides (−17 mg/dL, 95% CI −35, −0.19, *p* = 0.048 at 12 weeks). Adjusted between-group comparisons are provided in Table 3 [see also Supplementary Table S1].

Levels of HDL-C remained higher at both follow-up time points for the NUT2 arm compared to placebo (by approximately 5 mg/dL). However, these differences were not statistically significant.

Triglyceride levels, statistically lower for the NUT2 at baseline, continued to decrease with the differences between the two arms remaining statistically significant at both follow-up time points (*p* = 0.001 and *p* = 0.002, respectively).

#### 3.3.2. Homocysteine

Homocysteine concentrations were comparable between groups at baseline (NUT2: 9.38 ± 2.53 µmol/L; placebo: 8.92 ± 2.25 µmol/L; *p* = 0.5). By week 6, homocysteine had decreased substantially in the NUT2 group (7.86 ± 1.92 µmol/L) while remaining stable in the placebo group (9.14 ± 2.18 µmol/L), with a statistically significant between-group difference of −1.3 µmol/L (95% CI −2.1, −0.42; *p* = 0.004). At week 12, the divergence widened further: NUT2 7.27 ± 1.71 µmol/L versus placebo 9.29 ± 2.00 µmol/L, representing an adjusted between-group difference of −2.0 µmol/L (95% CI −2.8, −1.2; *p* < 0.001). This sustained reduction in homocysteine is consistent with the folic acid component of NUT2 and its established role in one-carbon metabolism (Figure 2c).

#### 3.3.3. Fasting Glucose and Insulin

**Figure 3:**
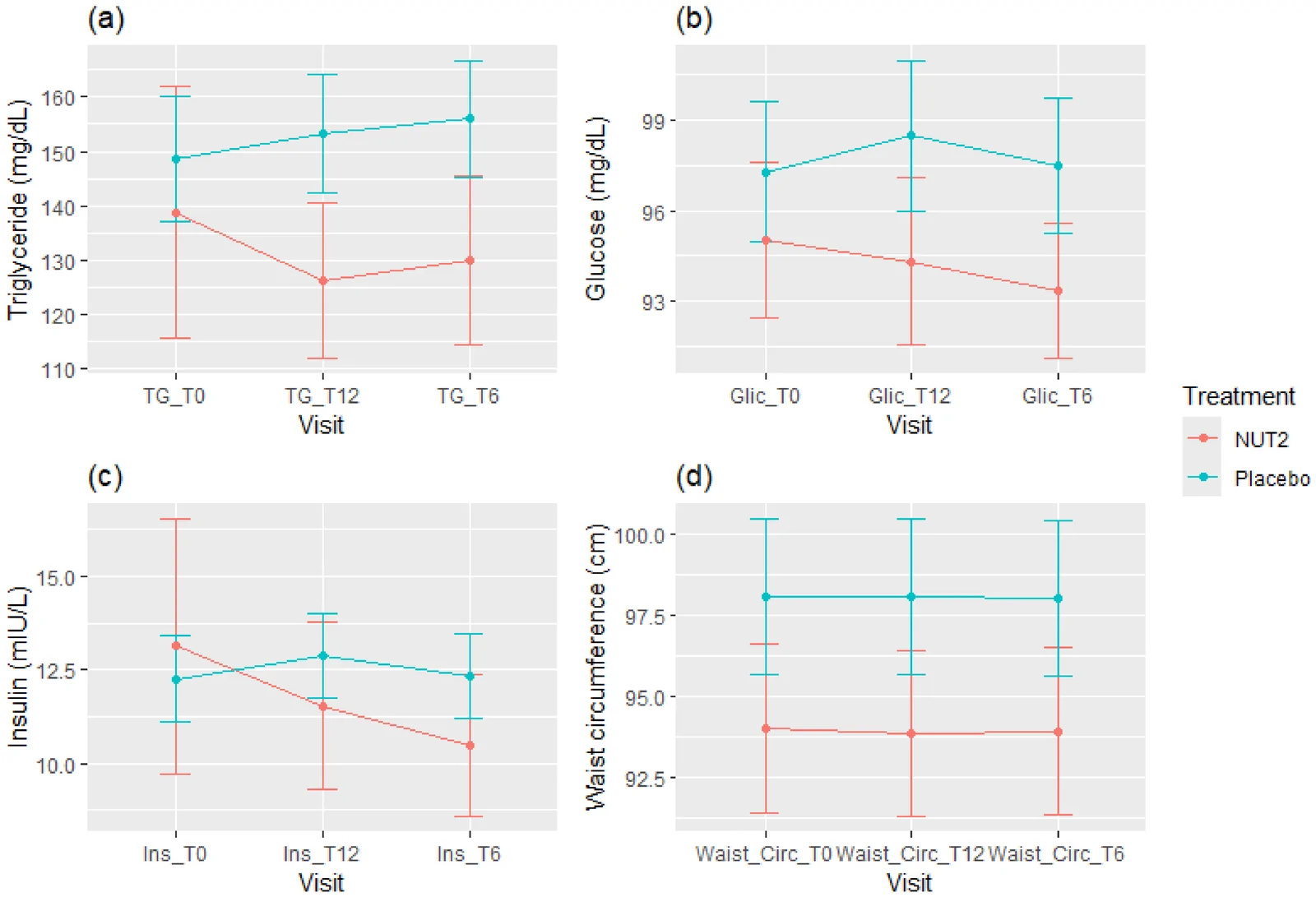
Composite metabolic outcome panel. Key: (**a**) triglyceride; (**b**) glucose; (**c**) insulin; (**d**) waist circumference.

Fasting plasma glucose remained largely stable in both groups throughout the study period (NUT2: baseline 95.0 ± 8.5 mg/dL; week 12: 94.3 ± 9.2 mg/dL; placebo: baseline 97.3 ± 7.7 mg/dL; week 12: 98.5 ± 8.4 mg/dL). Between-group differences were statistically significant at week 6 (*p* = 0.007) and week 12 (*p* = 0.003). However, the absolute difference in fasting glucose was small, and its clinical significance remains uncertain. Serum insulin decreased from 13.1 ± 11.4 mIU/L at baseline to 10.5 ± 6.4 mIU/L at week 6 in the NUT2 group, compared with minimal change in placebo (12.3 ± 3.9 to 12.3 ± 3.7 mIU/L). Fasting insulin concentrations were lower in the NUT2 group than in the placebo group during follow-up. The between-group difference was statistically significant at week 6 (*p* = 0.002) and remained so at week 12 (*p* = 0.014) (Figure 3a–d).

#### 3.3.4. Other Parameters

The differences between the two arms in terms of body weight remained consistent over time, with the NUT2 arm maintaining a lower mean body weight (by around 3 kg) relative to the placebo arm. These differences were not statistically significant (*p* = 0.3). A similar pattern was also observed for waist circumference, with the NUT2 maintaining a smaller waist circumference (approximately 4 cm) compared to placebo over time (Table 3). These differences were statistically significant (*p* < 0.05).

There were no meaningful differences between treatments for hsCRP at either week 6 or week 12, despite a small increase in mean hsCRP levels for the NUT2 arm by week 12 (*p* = 0.7).

#### 3.3.5. Metabolic Syndrome

Metabolic syndrome prevalence at baseline was 17.8% in the NUT2 group (8/45) and 28.9% in the placebo group (13/45; *p* = 0.2). At 12 weeks, the trajectories diverged markedly: metabolic syndrome prevalence decreased to 13.3% with NUT2, whilst increasing to 37.8% with placebo (*p* = 0.008).

**Figure 4:**
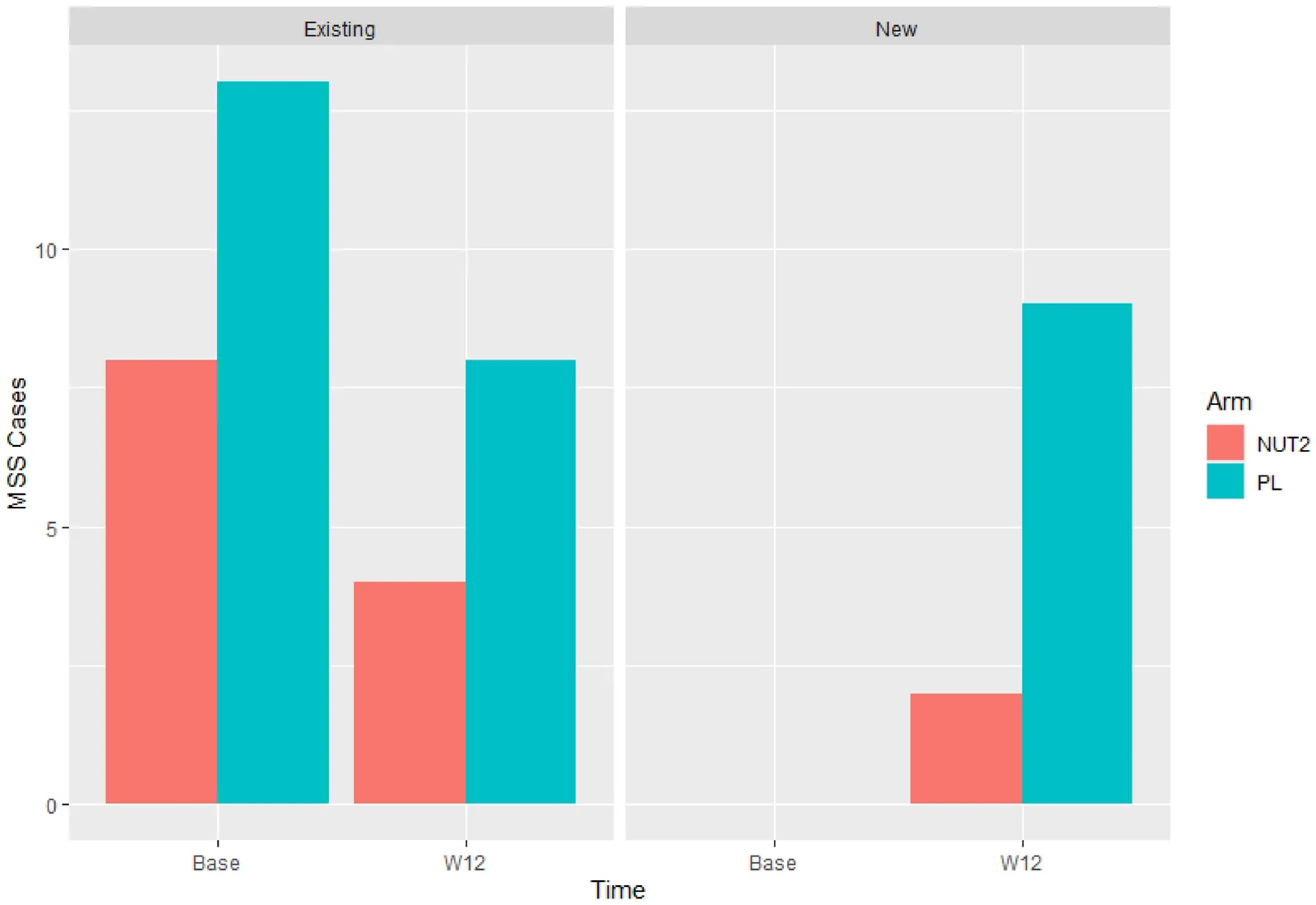
Metabolic syndrome prevalence bar chart.

Analysis of individual subject trajectories revealed that 50% of NUT2 participants with metabolic syndrome at baseline (4/8) had resolution of the diagnosis by week 12, compared with 23% in the placebo arm (3/13). Conversely, new metabolic syndrome emerged in 20% of NUT2 participants without the diagnosis at baseline (2/10), compared with 41% in the placebo group (9/22). These findings, whilst based on modest subgroup numbers, indicate that NUT2 was associated with both greater resolution of established metabolic syndrome and potential benefit against incident metabolic syndrome over the 12-week period (Figure 4).

### 3.4. Acceptability and Principal Investigator (PI) Evaluation

The PI’s final judgment of treatment efficacy was rated as good or very good in 71.1% of subjects receiving NUT2 compared with 22.2% receiving placebo (*p* < 0.001). Willingness to continue treatment was similarly higher among NUT2 subjects (86.7%) versus placebo (40.0%) (*p* < 0.001).

### 3.5. Safety and Adverse Events

NUT2 was generally well tolerated. One participant reported moderate leg cramps after 30–45 days, assessed as possibly related to the treatment. Symptoms resolved within 7–10 days after discontinuation. In the placebo group, one subject reported difficulty taking the tablets daily, and two reported a bitter taste and digestive discomfort. No serious adverse events occurred in either group.

## 4. Discussion

The present randomized, placebo-controlled study demonstrates that 12 weeks of supplementation with the multi-component nutraceutical NUT2 produced clinically meaningful reductions in LDL-cholesterol and total cholesterol in adults with low-to-moderate cardiovascular risk. These findings are particularly relevant in the context of contemporary European prevention frameworks, which increasingly recognise cardiovascular risk as a continuum and highlight the limitations of lifestyle intervention alone in a substantial proportion of primary prevention patients who do not yet meet criteria for pharmacological therapy [1].

The magnitude of LDL-C reduction observed with NUT2 was significant, with an unadjusted between-group difference of −10 mg/dL (95% CI −18, −2.1; *p* = 0.014) and a baseline-corrected difference of −23 mg/dL (95% CI −29, −16; *p* < 0.001) at 12 weeks. Both estimates substantially exceed the modest lipid changes typically achieved with dietary intervention alone, as documented in previous meta-analyses [7]. Importantly, the study population reflects a clinically common phenotype in which cardiometabolic risk is driven not by isolated hypercholesterolaemia but by the clustering of metabolic abnormalities. In this setting, interventions that address multiple interconnected pathways may be more appropriate than single-mechanism strategies.

The constellation of metabolic improvements observed across lipid, glycaemic and inflammatory domains is consistent with the complementary biological activities of the formulation components. However, because this study evaluated the proprietary formulation as a whole, the contribution of individual ingredients cannot be determined. The observed reduction in homocysteine is biologically plausible given the inclusion of folic acid, which plays an established role in one-carbon metabolism and homocysteine remethylation. Similarly, previous experimental and clinical studies have suggested that berberine and bergamot may influence lipid metabolism through complementary mechanisms. Nevertheless, the secondary biomarker findings should be regarded as exploratory and hypothesis-generating, and the underlying mechanisms require confirmation in dedicated mechanistic studies.

Beyond lipid parameters, NUT2 was associated with a clinically notable difference in metabolic syndrome trajectory over 12 weeks. Whilst metabolic syndrome prevalence decreased in the NUT2 group, it increased substantially in the placebo arm, a statistically significant between-group difference that reflects the cumulative effect of improvements across multiple metabolic domains rather than any single parameter. The resolution rate of established metabolic syndrome (50% in NUT2 vs. 23% in placebo) and the substantially lower incidence of new metabolic syndrome (20% vs. 41%) are noteworthy, though the absolute numbers are modest, and these findings should be considered hypothesis generating. Nonetheless, they are directionally consistent with the mechanistic profile of the formulation and suggest potential utility in modifying early cardiometabolic disease trajectory, a clinically meaningful goal within this population.

In contrast, no meaningful effect on hsCRP was observed. This likely reflects the relatively low baseline inflammatory burden of the study population and the limited duration of follow-up, rather than an absence of biological effect. Future studies enriched for participants with elevated inflammatory markers or with longer intervention periods may be required to fully evaluate potential anti-inflammatory benefits.

Treatment acceptability and investigator-rated efficacy were substantially higher in the NUT2 group. The favourable safety profile observed over the 12-week study period suggests that NUT2 was well tolerated and may represent a feasible adjunct to lifestyle intervention in appropriately selected primary prevention populations.

This study has limitations, including its relatively short duration, modest sample size and lack of long-term cardiovascular outcome data. An additional limitation is that adherence to the prescribed dietary intervention was assessed by participant self-report rather than by objective dietary assessment methods, such as food diaries or repeated dietary recalls. Although both study groups received identical dietary counselling and were randomised only after completing the same dietary run-in period, some residual confounding arising from unmeasured differences in dietary adherence between participants cannot be excluded. Consequently, attribution of the observed metabolic effects solely to the nutraceutical intervention should be made with appropriate caution. The dietary run-in period also represents a limitation of the study. Participants were randomised only if LDL-cholesterol remained inadequately controlled despite adherence to the prescribed diet. Although this design was intended to identify the patient population most likely to be considered for adjunctive nutraceutical therapy, it enriched the study cohort for individuals with limited responsiveness to diet alone. Consequently, the findings may not be generalisable to individuals who achieve substantial lipid improvements through lifestyle modification alone. One final limitation is that the trial was registered retrospectively rather than prospectively. Although the study protocol and predefined outcome measures were established before participant enrolment, retrospective registration is less consistent with current best practice for clinical trial transparency.

Nevertheless, the study provides clinically relevant evidence that a rationally designed nutraceutical combination can meaningfully improve lipid and metabolic profiles in low–moderate risk individuals who remain above optimal targets despite lifestyle intervention. These findings suggest that combination nutraceuticals may have a role as adjuncts to lifestyle intervention in appropriately selected individuals, although confirmation in larger and longer-term studies is required.

## 5. Conclusions

In this randomized, placebo-controlled study, NUT2 produced significant reductions in LDL-cholesterol and total cholesterol over 12 weeks in adults with low-to-moderate cardiovascular risk whose lipid levels remained suboptimal despite dietary intervention. Exploratory analyses also demonstrated favourable changes in several secondary metabolic biomarkers, including homocysteine and metabolic syndrome status.

The formulation was safe and well tolerated, with high treatment acceptability and investigator-rated efficacy, supporting its practical suitability for long-term use in primary prevention settings where sustained adherence is essential.

Collectively, these findings suggest that NUT2 may have potential as an adjunct to lifestyle intervention in individuals with low-to-moderate cardiovascular risk whose lipid levels remain suboptimal despite dietary modification. Exploratory findings also indicated favourable changes in selected metabolic biomarkers that require confirmation. Further studies of longer duration and in larger populations are warranted to assess the durability and clinical significance of these findings.

## Figures and Tables

**Table 1:** Baseline demographic and lifestyle details.

Characteristic	NUT-2 (*N* = 45)	Placebo (*N* = 45)	*p*-Value
**Gender**			
Female	29/45 (64.4%)	23/45 (51.1%)	0.2
Male	16/45 (35.6%)	22/45 (48.9%)	
**Age**	51.40 (11.26)	52.38 (9.50)	0.7
**BMI (kg/m^2^)**	24.67 (3.29)	25.48 (2.97)	0.2
**Waist circumference (cm)**	94.00 (8.73)	98.09 (8.03)	0.03
**Smoking history**			
Former smoker	6/45(13.3%)	3/45 (6.7%)	0.5
Never smoked	23/45 (51.1%)	28/45 (62.2%)	
Current smoker	16/45 (35.6%)	14/45 (31.1%)	
**Familial cardiovascular disease**			
No	33/44 (75.0%)	33/44 (75.0%)	>0.9
Yes	11/44 (25.0%)	11/44 (25.0%)	
**Physical activity**			
No	23/44 (52.3%)	20/42 (47.6%)	0.7
Yes	21/44 (47.7%)	22/42 (52.4%)	
**Alcohol consumption**			
None	18/45 (40.0%)	13/41 (31.7%)	0.4
Normal	27/45 (60.0%)	28/41 (68.3%)	
**Diabetes**			
No	45/45 (100.0%)	44/44 (100.0%)	

**Table 2:** Baseline clinical variables.

Characteristic	NUT-2	Placebo	*p*-Value
**Anthropometric and Haemodynamic Parameters**			
Weight (kg)	71.16 (10.99)	74.31 (12.91)	0.3
SBP (mmHg)	121.22 (7.62)	123.78 (7.00)	0.042
DBP (mmHg)	77.56 (5.29)	79.00 (5.60)	0.2
HR (bpm)	71.62 (8.24)	67.73 (5.84)	0.005
**Cardiovascular Risk Markers**			
Total cholesterol (mg/dL)	237.31 (27.17)	222.89 (17.92)	0.006
LDL cholesterol (mg/dL)	150.47 (20.74)	138.02 (15.05)	0.003
HDL cholesterol (mg/dL)	60.27 (13.15)	55.42 (8.57)	0.089
Triglycerides (mg/dL)	138.76 (77.43)	148.51 (38.23)	0.042
Glucose (mg/dL)	95.02 (8.48)	97.27 (7.73)	0.13
Homocysteine (μmol/L)	9.38 (2.53)	8.92 (2.25)	0.5
**Metabolic syndrome**			
No	37.0/45.0 (82.2%)	32.0/45.0 (71.1%)	0.2
Yes	8.0/45.0 (17.8%)	13.0/45.0 (28.9%)	
**Metabolic and Inflammatory Markers**			
hsCRP (mg/L)	1.42 (1.23)	1.65 (1.34)	0.4
Insulin (mIU/L)	13.13 (11.36)	12.26 (3.90)	0.3

**Table 3:** Lipid parameters at baseline and Weeks 6 and 12.

Changes Corrected by Baseline Score	Baseline—Week 6								Baseline—Week 12							
Characteristic	NUT2	NUT2% Change	PL	PL % Change	Difference	95% CI	*p*-Value	NRR (%)	NUT2	NUT2% Change	PL	PL % Change	Difference	95% CI	*p*-Value	NRR (%)
Total cholesterol (mg/dL)	−14.24 (23.68)	−6	1.84 (7.28)	0.826	−16	−23, −8.7	<0.001	−6.826	−23.98 (29.69)	−10.1	2.36 (9.04)	1.059	−26	−36, −17	<0.001	−11.16
LDL cholesterol (mg/dL)	−14.84 (20.85)	−9.86	0.73 (6.91)	0.529	−16	−22, −9.0	<0.001	−10.39	−20.51 (18.34)	−13.63	2.07 (9.27)	1.5	−23	−29, −16	<0.001	−15.13
HDL cholesterol (mg/dL)	1.20 (4.58)	1.99	0.80 (3.45)	1.444	0.4	−1.3, 2.1	0.6	0.55	−0.53 (6.06)	−0.83	−0.53 (3.60)	−0.902	0	−2.1, 2.1	>0.9	0.07
Triglycerides (mg/dL)	−8.76 (49.96)	−6.31	7.47 (21.64)	5.03	−16	−32, 0.01	0.05	−11.34	−12.67 (55.48)	−9.13	4.78 (15.73)	3.219	−17	−35, −0.19	0.048	−12.35
Homocysteine (µmol/L)	−1.53 (1.48)	−1.1	0.22 (1.14)	0.148	−1.7	−2.3, −1.2	<0.001	−1.25	−2.11 (1.69)	−1.52	0.37 (1.10)	0.249	−2.5	−3.1, −1.9	<0.001	−1.77
Glucose (mg/dL)	−1.69 (6.79)	−1.22	0.20 (3.88)	0.135	−1.9	−4.2, 0.44	0.11	−1.36	−0.71 (8.62)	−0.51	1.20 (4.98)	0.808	−1.9	−4.9, 1.0	0.2	−1.32
Insulin (mIU/L)	−2.65 (8.98)	−1.91	0.06 (1.42)	0.04	−2.7	−5.4, 0.02	0.052	−1.95	−1.60 (8.39)	−1.15	0.61 (1.58)	0.411	−2.2	−4.8, 0.35	0.089	−1.56
Waist circumference (cm)	−0.09 (0.36)	−0.06	−0.07 (0.33)	0	−0.02	−0.17, 0.12	0.8	−0.06	−0.16 (0.47)	−0.12	−0.02 (0.45)	0	−0.13	−0.33, 0.06	0.2	−0.12
Weight (kg)	−0.20 (0.59)	−0.14	0.02 (0.62)	0.013	−0.22	−0.48, 0.03	0.085	−0.15	−0.40 (0.75)	−0.29	0.13 (0.79)	0.088	−0.53	−0.86, −0.21	0.001	−0.38

Key: NRR, net relative reduction.

## Data Availability

The data presented in this study are available on request from the corresponding author due to commercial restrictions.

## Supplementary Materials

The following supporting information can be downloaded at: https://www.mdpi.com/article/10.3390/nu18162632/s1, Table S1: Other parameters by study visit.
