# Supplementary material for: Effects of a Multi-Component Nutraceutical (NUT2) on the Lipid Profile in Adults with Low-to-Moderate Cardiovascular Risk: A Randomized Placebo-Controlled Trial
Source: Nutrients. 2026 Aug 12;18(16):2632. doi: 10.3390/nu18162632 (PMC13517055; doi:10.3390/nu18162632)
Supplement: Supplementary file 1 [file nutrients-18-02632-s001.zip › nutrients-4440084-supplementary.pdf]

Supplementary Table S1. Other parameters by study visit

Characteristic	NUT2	Placebo	p
**Waist circumference baseline (cm)**	94.00 (8.73)	98.09 (8.03)	0.03
**Waist circumference Week 6 (cm)**	93.91 (8.64)	98.02 (7.96)	0.028
**Waist circumference Week 12 (cm)**	93.84 (8.62)	98.07 (8.00)	0.026
**Insulin baseline (mIU/L)**	13.13 (11.36)	12.26 (3.90)	0.3
**Insulin Week 6 (mIU/L)**	10.48 (6.39)	12.32 (3.74)	0.002
**Insulin Week 12 (mIU/L)**	11.53 (7.47)	12.87 (3.81)	0.014
**hsCRP baseline (mg/L)**	1.42 (1.23)	1.65 (1.34)	0.4
**hsCRP Week 6 (mg/L)**	1.53 (1.16)	1.56 (1.16)	0.8
**hsCRP Week 12 (mg/L)**	2.21 (4.08)	1.49 (1.00)	0.7
